# Early prognostic performance of miR155-5p monitoring for the risk of rejection: Logistic regression with a population pharmacokinetic approach in adult kidney transplant patients

**DOI:** 10.1371/journal.pone.0245880

**Published:** 2021-01-22

**Authors:** Luis Quintairos, Helena Colom, Olga Millán, Virginia Fortuna, Cristina Espinosa, Lluis Guirado, Klemens Budde, Claudia Sommerer, Ana Lizana, Yolanda López-Púa, Mercè Brunet

**Affiliations:** 1 Faculty of Pharmacy and Food Sciences, Biopharmaceutics and Pharmacokinetics Unit, Department of Pharmacy, Pharmaceutical Technology and Physical Chemistry, University of Barcelona, Barcelona, Spain; 2 Pharmacology and Toxicology Laboratory, Biochemistry and Molecular Genetics, Biomedical Diagnostic Center (CDB), Hospital Clinic of Barcelona, University of Barcelona, Barcelona, Spain; 3 Biomedical Research Center in Hepatic and Digestive Diseases (CIBERehd), Instituto de Salud Carlos III, Madrid, Spain; 4 Renal Transplant Unit, Nephrology Department, Fundació Puigvert, Barcelona, Spain; 5 Medizinische Klinik mit Schwerpunkt Nephrologie, Charité Universitätsmedizin Berlin, Berlin, Germany; 6 Department of Nephrology, University of Heidelberg, University Hospital of Heidelberg and Mannheim, Heidelberg, Germany; 7 Quality Unit of CDB, Hospital Clinic of Barcelona, University of Barcelona, Barcelona, Spain; Medical University of Gdansk, POLAND

## Abstract

Previous results from our group and others have shown that urinary pellet expression of miR155-5p and urinary CXCL-10 production could play a key role in the prognosis and diagnosis of acute rejection (AR) in kidney transplantation patients. Here, a logistic regression model was developed using NONMEM to quantify the relationships of miR155-5p urinary expression, CXCL-10 urinary concentration and tacrolimus and mycophenolic acid (MPA) exposure with the probability of AR in adult kidney transplant patients during the early post-transplant period. Owing to the contribution of therapeutic drug monitoring to achieving target exposure, neither tacrolimus nor MPA cumulative exposure was identified as a predictor of AR in the studied population. Even though CXCL-10 urinary concentration showed a trend, its effect on AR was not significant. In contrast, urinary miR155-5p expression was prognostic of clinical outcome. Monitoring miR155-5p urinary pellet expression together with immunosuppressive drug exposure could be very useful during routine clinical practice to identify patients with a potential high risk of rejection at the early stages of the post-transplant period. This early risk assessment would allow for the optimization of treatment and improved prevention of AR.

## Introduction

Despite advances in immunosuppression, allograft rejection and clinical outcomes remain a challenge in solid organ transplantation. The dosages of immunosuppressive drugs (ISDs) are adjusted to achieve target concentrations and to prevent rejection or drug-related adverse events. The combined pharmacokinetic monitoring of ISDs and immunological biomarkers may provide patient risk stratification and allow for personalized therapy. The results from several studies demonstrate the need to identify and validate noninvasive prognostic and diagnostic biomarkers for allograft rejection and to monitor the allograft condition at early time points with the aim to achieve personal therapy adjustment before graft injury occurs [1–3].

In a previous study on kidney transplant recipients [4], our group identified miR155-5p and interferon gamma inducible chemokine 10 (CXCL-10) as prognostic and diagnostic biomarkers of rejection based on a receiver operating characteristic (ROC) curve data analysis. These results are in agreement with those reported by *Wilflingseder* et al. and *Soltaninejad* et al. in kidney transplant recipients [5,6].

MicroRNAs (miRs) are endogenous noncoding RNA molecules that regulate gene expression [7] at the post-transcriptional level and are involved in regulating several aspects of inflammation [8] as well as affecting the immune response [9]. miR155-5p is a mediator of the inflammatory response and a regulator of IFN-γ production in T-cells and NK-cells. This miR participates in the regulation of adaptative immunity and antibody-related T-cell responses.

CXCL-10 is predominantly secreted by leukocytes in transplanted allografts and tubular epithelial cells, and it binds CXC receptor 3 (CXCR3), which is predominantly expressed on T lymphocytes [10]. CXCL-10 has been identified as a long- and short-term graft function [11] and injury [12] biomarker in kidney transplant recipients.

In these previous studies, data analyses for drug exposure and biomarkers and their correlation with rejection did not consider the most appropriate models, such as logistic regression models [13], by means of the population approach.

This study aimed to develop a logistic regression model to investigate whether urinary miR155-5p pellet expression and urinary CXCL-10 concentration may predict the probability of rejection in adult kidney transplant recipients. Moreover, the utility of measuring tacrolimus and mycophenolic acid (MPA) cumulative exposures to reduce the risk of rejection was also evaluated.

## Material and methods

### Ethical conduct of the study

The study was approved by the ethics committees of all participating centers (Charité: Ethik-Kommission des Landes Berlin Landesamt für Gesundheit und Soziales Berlin Fehrbelliner Platz 1 10707 Berlin; Heidelberg: Ethik-Kommission der Medizinischen Fakultät Heidelberg. Alte Glockengießerei 11/1 D-69115 Heidelberg;Puigvert: Comité Ético de Investigación Clínica (CEIC) Fundación Puigvert). All patients provided their written informed consent prior to their inclusion in the study. In all centers, the medical costs were covered according to national health insurance regulations. No cash payments were provided to donors or donors’ families.

### Population

In a European multicenter prospective observational study coordinated and performed by our group (*EudraCT number*: 2013-001817-33), 80 de novo adult kidney recipients from deceased or living donor patients were recruited between 2014 and 2015 by three European centers: Charité Universitäts Medizin, Berlin, (Germany), Universitätsklinikum, Heidelberg, (Germany) and Fundació Puigvert, Barcelona, (Spain) [4]. Living donors (relatives or well-known donors) had undergone a strict evaluation both of the medical and psychological status according to the guidelines and by the ethical board of each center. The cause of death of deceased donors did not involve kidney injury but traumatic brain injury, hemorrhagic stroke, ischemic stroke, cerebral anoxia or non-heartbeating, or controlled asystolia; these donors were included through Eurotransplant regular allocation process.

Data from 58 out of 80 patients were included in the pharmacokinetic sub-study. Some of the planned samples to be collected according to the protocol could not be obtained for different reasons, all of them related to unavoidable difficulties frequently arising in the clinical practice such as clotted blood samples, hemolysis occurrence, no attendance of patient to the visit, difficulties in the blood extraction or sample loss during the analytical procedures, among others. This issue led to less available tacrolimus and MPA concentration-time data for the PK/PD analysis. Recipients who were older than 70 years old, hepatitis B or C positive, human immunodeficiency virus (HIV) positive, or combined liver-kidney recipients were excluded. The study was intended to cover the early post-transplantation stages spanning the first 6 months. Patients had a total of five visits: 1^st^ week, 1^st^ month, 2^nd^ month, 3^rd^ month and 6^th^ month after transplantation. The day of the 1^st^ week visit varied from 1 to 11 days posttransplant. The diagnosis of acute rejection (AR) was based on renal function impairment detected by creatinine concentrations and glomerular filtration rate (GFR) by the modification of Diet in Renal Disease (MDRD) method [14]. GFR was stratified based on Kidney Disease Outcomes Quality Initiative guideline for evaluation, classification, and stratification of chronic kidney disease (KDOQI CKD) classification as: chronic dialysis or need for re-transplantation as end-stage kidney-failure; GFR<29 as severe decrease in renal function; 30<GF<59 as moderate decrease in kidney function; and 60<GF<90 mild decreases in kidney function. A histological evaluation of graft biopsy (biopsy-proven acute rejection [BPAR]) according to the Banff 2011 [14] evaluation was used to confirm AR occurrence.

### Treatment, blood sampling and analytical methods

#### Immunosuppressive treatment

All patients received the same immunosuppressive therapy according to the clinical protocol of the IMAGEN study [4] and in line with the local standard of care. Immunosuppressive treatment consisted of tacrolimus (Prograf^®^, Astellas Pharma), mycophenolate mofetil (Myfenax^®^, Teva Pharmaceuticals), and methylprednisolone. All patients received an induction treatment of two 20-mg doses of basiliximab. From each patient, blood samples were collected at 0, 0.5, 1, 1.5, 2, 3, 4, 6, 8 and 12 hours after drug administration in the 1^st^ week and at 0, 1.5, 2 and 4 hours after drug administration in the 1^st^, 2^nd^, 3^rd^ and 6^th^ months after transplantation. Drug analysis was performed in the respective centers involved in the study. Tacrolimus concentrations were measured in whole blood by liquid chromatography/tandem mass spectrometry, whereas MPA concentrations were determined in plasma by high-performance liquid chromatography with ultraviolet detection [15–17].

#### Immunological biomarkers

First morning urine samples for biomarkers analysis were withdrawn at the 1^st^ week and the 1^st^, 2^nd^, 3^rd^ and 6^th^ months after transplantation. All samples were shipped to the Pharmacology Laboratory of the Biomedical Diagnostic Center, Hospital Clinic (Barcelona, Spain) for a centralized blinded fashion analysis.

CXCL-10 and miR155-5p were evaluated following the methods described in our previous publication [4]; briefly, miR155-5p expression was analyzed from urinary pellets by quantitative polymerase chain reaction (qPCR) as follows. Urine specimens were collected in the presence of EDTA RNAse at 4°C. RNA was extracted using TRIzol™ reagent (Life Technologies), according to the manufacturer’s instructions. Total RNA was reverse transcribed into cDNA, and qPCR was performed using the miRCURY LNA^TM^ Universal RT microRNA PCR, Polyadenylation and cDNA synthesis system (Exiqon, Denmark). Cq values for all samples were determined, and the ΔCq was calculated as the difference in Cq values between the miRNA target and the reference control. Relative expression levels of target miRNAs were then evaluated within a sample according to the formula 2^-ΔCq^, where high values corresponded to higher expression.

Urinary CXCL-10 concentrations were analyzed using a commercial enzyme-linked immunosorbent assay kit (R&D Systems, Minneapolis, MN, USA) following the manufacturer’s recommendations. Urine samples were centrifuged at 3000 rpm for 10 min, and the supernatant was stored at -70°C. All samples were processed in duplicate. The minimum detectable concentration of CXCL-10 was 1.67 pg/mL.

### Data analysis

#### Exploratory statistical analysis

A previous exploratory statistical analysis was performed by comparing the mean values of trough concentrations and normalized to the dose trough concentrations for each ISD between occasions (from week 1 to month 6) and between patients who presented AR and those who did not over the period from week 1 to month 1 during which more rejections occurred. For that purpose, a two-way analysis of variance followed by the Tukey’s multiple comparison test was applied. In the first analysis, the occasion was considered as a fixed factor and the patient as a random factor. In the second case, the clinical outcome (AR versus non rejection) was taken as a fixed factor and the patient as a random factor nested within the clinical outcome variable.

#### Log-transformed data were used in all the cases

The significance was set at α = 0.05, and the SPSS version 25 statistical package was used.

### Pharmacokinetic-pharmacodynamic modeling

A sequential population pharmacokinetic–pharmacodynamic analysis was performed. First, all concentration versus time data of either tacrolimus or MPA were simultaneously analyzed, and their respective population pharmacokinetic models were developed. Then, the final pharmacokinetic parameters were fixed in the model, and a logistic regression was built between the individual predicted immunosuppressive drug (ISD) exposure values, observed biomarker urinary expression levels and transplant rejection outcomes. Of note, for modeling purposes, mycophenolate mofetil doses were transformed to MPA molar equivalents.

The nonlinear mixed effects models implemented in NONMEM version 7.4.1 software (ICON Development Solutions, Ellicott City, MD, USA) were used. Phoenix WinNonlin^®^ 6.4 [18], R 3.4.1 [19], Xpose 4.0 [20] R package, the visual predictive check (vpc) [21] R package, and Perl-speaks-NONMEM (psn) 5.3.2 [22] were used for graphical diagnostics and model evaluation. Pirana software [23] was used as a support tool throughout all building processes.

#### Pharmacokinetic models

*Base model*. In all the cases, the stochastic approximation expectation maximization estimation method (SAEM) that provides the population parameters converging toward the maximum of exact likelihood was used during all model building processes. Because the OFV given by SAEM does not enable the assessment of minimization due to its stochastic characteristics, the importance sampling (IMP) method was used for relative standard errors and objective function evaluation after the stochastic portion was completed [24]. MU-referenced coded parameters were used, as recommended, to gain efficiency [25].

One- and two-compartment models with linear elimination and first-order absorption kinetics were tested. The modeling of enterohepatic circulation was also attempted on MPA data, as previously reported [26]. The models were parameterized in terms of total clearance (CL), absorption rate constant (Ka), apparent distribution volumes (V_*n*_) and intercompartmental clearance (Q). Lag time was modeled using the classical approach as well as the transit compartments models (or erlang distribution) [27]. Distribution volumes and flow parameters were apparent values, i.e., (CL/F), (V_*n*_/F), (Q/F), because only concentrations after oral administration were available. All disposition pharmacokinetic parameters were a priori scaled with body-weight according to the allometry laws as proposed previously [28]. Fixed allometric exponents of 0.75 and 1 were applied to flow parameters and distribution volumes, respectively. This method is the standard and more parsimonious approach to report PK parameters to understand differences between humans of all ages. Although more physiological approaches are frequently used, no evidence exists that a significative improvement of the fit can be achieved with respect to the standard approach of fixed allometric exponent of 0.75 for CL [29].

Between-subject variability (BSV) and interoccasion variability (IOV) [30] were both tested in all parameters and described using exponential error models. First, the diagonal omega matrix of the interindividual random effects was evaluated; then, the omega block structure was explored, and the potential correlations among all the empirical Bayes estimates of the interindividual random effects were examined. Omega block structure was retained if correlations were found. Residual variability modeling was tested with untransformed and log-transformed concentration data. Additive, proportional and combined (additive-proportional) residual error models were tested for untransformed data and additively based on log-transformed data if proceeded [31].

*Covariate model*. Once the base models were achieved, the individual predicted parameters estimated from the population parameters and the empirical Bayes estimates of the interindividual random effects provided by SAEM were plotted versus all of the most physiologically meaningful available covariates, i.e., age, sex, donor type (cadaveric or living), glomerular filtration rate (GFR), donor age, cold ischemia time, time of dialysis, lymphocyte count and the occurrence or absence of diabetes mellitus during the study to identify potential relationships. The influence of dose was also investigated on CL/F and Vc/F to identify possible non-linear behaviors either associated with the elimination process or erythrocytes (tacrolimus) or protein binding (MPA). Then, the influence of all of the covariates was tested on the corresponding pharmacokinetic parameters by means of exponential, power and linear relationships. The covariates were introduced first one at a time and then sequentially according to the stepwise forward inclusion-backward elimination procedures [32].

#### Logistic regression model

A logistic regression model [33] was developed using the Laplacian first-order conditional estimation method. Treated as a binary variable, AR occurrence was used as a response variable, with 0 indicating no event and 1 indicating AR. For modeling purposes, rejection events were considered on the visit prior to their occurrence to evaluate the prognostic capacity of the explanatory variables. The individual predicted exposures to tacrolimus and MPA (given by either the cumulative area under the curve [AUC] or mean trough concentrations) and the observed urinary expression of miR155-5p and CXCL-10 were evaluated as possible factors influencing the response or explanatory variables. Individual cumulative exposure values were predicted with NONMEM by integrating the drug amounts in a “dummy” compartment according to the following equation:

$$AUC=\int_{0}^{t}C\cdot dt$$ (1)

The probability of AR occurrence was linked to explanatory variables through the logit transformation to ensure that the estimated probability fell between 0 and 1. Given that P_i_ is the probability of achieving an AR event, the model had the following structure:

$$LOGIT(Pi)=Ln\left(\frac{P_{i}}{1-P_{i}}\right)=\theta_{o}+\theta_{i}*X_{i}$$ (2)

$$P_{i}=\left(\frac{{exp}^{\theta_{o}+\theta_{i}*X_{i}}}{\left(1+{exp}^{\theta_{0}+{}_{i}*X_{i}}\right)}\right)$$ (3)

where θ_0_ is the baseline or intercept term, θ_i_ is the coefficient corresponding to the effect induced by Xi, where Xi is a given explanatory variable i (i = from 1 to n).

The same covariates considered in the pharmacokinetic models’ development were evaluated in the logistic regression as additional explanatory variables.

#### Model selection

At all of the stages of model building, the goodness-of-fit of the models to the data was evaluated as follows. To statistically distinguish between nested models, the likelihood ratio test, based on a reduction in the minimum objective function value (MOFV), was used (ΔMOFV: - 2 log likelihood, approximately χ^2^ distribution). A significance level of *p*<0.005 corresponding to a ΔMOFV = -7.879 for one degree of freedom was used. For nonhierarchical models, the most parsimonious model with the lowest MOFV according to the Akaike information criterion (AIC) was chosen. A decrease in MOFV ≥ 3.841 units (*p*<0.05) was considered to retain a covariate in the model during the forward inclusion, whereas an increase in MOFV ≥10.8 units (*p*<0.001) was considered to retain a covariate during the backward elimination. Moreover, the stability of the developed models was explored by examination of the covariate matrix of the estimates to verify that no high correlation between parameters existed. A condition number less than 1000 was always considered as a criterion of non-ill conditioning.

The parameter precision expressed as the relative standard error (RSE%), reduction in BSV associated with the parameters, model completion status, and visual inspection of goodness-of-fit plots were also considered for model selection. Diagnostic plots of observed data versus population predicted and individual predicted concentrations were evaluated for randomness around the identity line. Plots of conditional weighted residuals (CWRES) versus time and individual weighted residuals (IWRES) versus individual predictions were evaluated for randomness around zero. η- and ε-shrinkage values (when appropriate) [34], were assessed to know the feasibility of using the individual predicted parameter estimates by the models for model diagnostics.

#### Model evaluation

Once the final models were developed, the corresponding predictive capability was evaluated using a prediction-corrected visual predictive check (VPC) for continuous data in the case of the population pharmacokinetic models and for categorical data for the logistic regression models [35]. In both cases, 1000 simulations of the respective entire original datasets including patient characteristics, dosing and sampling times were obtained, and the distributions for observed and simulated data were compared [35]. Nonparametric bootstraps were also performed to evaluate model stability and parameter precision. The adequacy of the PK models was assessed through the inverse cumulative density function and normalized prediction distribution errors (npde) [30]. Additionally, in the case of logistic regression, a grouped-bar graphic was constructed by plotting the proportion of the observed and predicted values of rejections versus the explanatory variable values, separated into 10 bins.

## Results

### Population characteristics and clinical outcome

All patient baseline demographic, biochemical and clinical characteristics are summarized in Table 1. Eight patients out of 58 (14%) developed at least one AR episode during the study. The observed eight rejections were cellular rejections and were diagnosed by a histological evaluation of graft biopsies (BPAR). Four episodes of rejection occurred during the 1st week after transplantation, three at the end of the 1st month, and one during the 6th month. In all patients who developed at least one AR event, urine for biomarker analysis was collected before the immunosuppressive treatment modification to resolve the AR episode [4]. All patients that rejected during the earliest post-transplant stages (week 1-month 1) had GFR <25 ml/min except one. At these stages, the GFR for the patient that rejected at month 6 ranged from 9.25 to 25.22 mL/min.

**Table 1:** Patient baseline, demographic, biochemical and clinical characteristics.

	Units	Median (IQR**)
Age	years	48 (38–58)
Donor Age	years	52 (45–60)
Sex (female/male)	n	20/38
Donor type (living/cadaveric)	n	30/28
Weight	kg	73 (62.9–86.8)
Height	cm	170 (163–177)
Body mass index	kg/m^2^	24 (22–29)
Glomerular filtration rate	mL/min	44 (15–55)
Infection occurrence	n (%) *	34 (59%)
Acute rejection occurrence	n (%) *	8 (14%)
Cytomegalovirus	n (%) *	14 (24%)
BK virus	n (%) *	9 (15%)
Diabetes mellitus	n (%) *	5 (9%)

* Percentages estimated with respect to the total number of patients included in the population PK analysis, N = 58.

**IQR: inter-quartile range (25th and 75th percentiles).

### ISDs, exposures and biomarker expression levels

Neither ISDs nor biomarkers showed concentrations below the limit of quantification [4]. Mean global and per occasion values of observed trough concentrations for ISDs and for biomarker expression levels are summarized in Table 2. In general, for both ISDs, mean trough concentrations tended to increase from week 1 to month 1; higher values compared with week 1 were maintained from month 2 to 3, and a slight decrease was observed at month 6, likely due to dose reductions that occurred from months 3 to 6. No significant differences were found in trough concentrations of MPA between any of the occasions (from week 1 to month 6). Conversely, significant differences were found between tacrolimus trough concentrations at month 1 and those of the other occasions. Normalized by dose, trough concentrations tended to increase with post-transplant time. Specifically for MPA, a significant increase was observed from week 1 to month 1, and then exposure values were maintained similar. By contrast, for tacrolimus normalized by dose trough concentrations increased along all post-transplant periods.

**Table 2:** Global and per occasion mean values (IQR) of ISDs trough concentrations and biomarkers’ expression.

Parameters	Units	Mean* (IQR**)					
		Global	Week 1	Month 1	Month 2	Month 3	Month 6
***Tacrolimus***							
**Dose**	**mg**	8.666 (6–10)	14.60 (13–18.5)	10.63 (9–16)	7.78 (6–11)	6.79 (5–10)	5.29 (4–7.75)
**Ctrough**	**ng/mL**	9.26 (7.4–12.0)	8.85# (6.49–11.95)	11.15 (8.78–14.17)	8.92# (7.10–12.41)	9.11# (7.67–11.06)	8.35# (7.22–10.46)
**Ctrough/Dose**	-	1.28 (0.73–1.66)	0.61 (0.43–0.85)	1.05## (0.76–1.4)	1.14## (0.85–1.62)	1.36##& # (1.00–1.82)	1.63##&# (1.15–2.19)
***MPA***							
**Dose**^**$**^	**mg**	1546.77 (1250–2000)	1875.91 (2000–2000)	1655.41 (1500–2000)	1552.02 (1250–2000)	1402.64 (1000–2000)	1238.67 (1000–2000)
**Ctrough**	**μg/mL**	2.64 (1.76–3.90)	2.37 (1.44–3.88)	2.93 (1.91–4.46)	2.70 (1.92–3.51)	2.90 (1.99–4.35)	2.37 (1.64–3.59)
**Ctrough/Dose**^**$**^	**-**	0.0021 (0.0013–0.0025)	0.0013 (0.0008–0.002)	0.0018## (0.0011–0.0026)	0.0017 (0.0014–0.0026)	0.0021## (0.0014–0.0031)	0.0019## (0.0015–0.0024)
***Biomarkers***							
**miR155-5p**	**ΔCt**	0.39 (0.03–0.55)	0.08 (0.03–0.23)	0.20## (0.05–0.96)	0.21## (0.06–0.81)	0.12 (0.04–0.40)	0.14 (0.04–0.61)
**CXCL-10**	**pg/mL**	81.63 (22.26–107.55)	73.81 (37.98–144.49)	38.04## (18.59–72.44)	36.65## (15.81–66.12)	43.90## (23.65–86.83)	43.66## (20.19–107.53)

* Mean expressed as geometric mean.

**IQR: inter-quartile range (25th and 75th percentiles).

^$^ MMF daily dose of Myfenax.

# *p*<0.05 comparisons versus month 1.

## *p*<0.05 comparisons versus week 1 & *p*<0.05 comparisons versus month 2.

Regarding biomarker expression levels, miRNA155 expression increased from week 1 to months 1–2, and then, a slight decrease occurred. CXCL-10 expression decreased from week 1 up to month 2 and stabilized in months 3 and 6. In both cases, significant differences were observed between week 1 and month 1 and on some of the other occasions (month 2 for miRNA155 and months 2, 3, and 6 for CXCL-10).

Comparative mean values of trough concentrations in patients who underwent AR versus those that who did not at the earliest post-transplant stages are displayed in Table 3. A trend toward higher values of tacrolimus trough concentrations in patients who did not develop AR versus those who did (*p* = 0.048) was evident, while the opposite trend was observed for MPA, but without a significant difference (*p*>0.05). By contrast significant differences were found between the expression of miRNA155 (*p*<0.001) and CXCL-10 (*p* = 0.004) in both groups, with higher levels of both biomarkers in patients who presented AR compared with those who did not.

**Table 3:** Mean values (IQR) of ISDs trough concentrations and biomarker expression levels for patients who presented AR and patients who did not from week 1 to month 1.

Parameters	Units	Mean* (IQR**)	
		Patients with AR	Patients without AR
***Tacrolimus***			
**Dose**	**mg**	9.12 (8.5–14.5)	12.89 (10–16)
**Ctrough**	**ng/mL**	7.37 (5.10–10.95)	10.10 (8.00–13.20)
**Ctrough/Dose**	**-**	1.18 (0.43–1.52)	0.90 (0.53–1.10)
***MPA***			
**MMF Dose** ^**$**^	**mg**	2000 (2000–2000)	1740.9 (1500–2000)
**Ctrough**	**μg/mL**	3.09 (2.55–5.13)	2.57 (1.58–3.97)
**Ctrough/Dose**^**$**^	**-**	0.0018 (0.0013–0.0026)	0.0019 (0.001–0.002)
***Biomarkers***			
**miR155-5p**	**ΔCt**	1.50 (1.15–1.85)	0.08 (0.03–0.23)
**CXCL10**	**pg/mL**	171.2 (148.09–206.64)	47.2 (23.83–92.52)

* Mean expressed as geometric mean.

**IQR: inter-quartile range (25th and 75th percentiles).

^$^ MMF daily dose of Myfenax.

Among patients who experienced rejection, only two had tacrolimus trough concentrations below the recommended acceptable range (4 ng/mL– 11 ng/mL) [3] at the earliest post-transplant stages (week 1-month 1); for two other patients, not coincident with the previous, MPA trough concentrations were under the recommended therapeutic range (< 1.6 ng/mL) [36]. Of note, MPA concentrations lower than 1.6 ng/mL were also observed at week 1 in 17 patients who did not develop acute rejection.

### Population pharmacokinetic models

#### Tacrolimus

A total of 1102 concentration time points from 58 patients were included in the analysis. A two-compartment model with lag time, first-order absorption and first-order elimination fit the data best. A log transformation of the data stabilized the model. BSV was only be associated with the whole blood clearance (CL), intercompartmental clearance (Q) and central volume of distribution (V_C_).

Residual error was better described by an additive model on log-transformed data. The final estimated PK parameters and their precisions (RSE), BSV effect on each parameter and η-shrinkage and ε-shrinkage values were acceptable and are listed in Table 4.

**Table 4:** Estimated parameters and bootstrap results of the final population PK models.

		**Tacrolimus**			**MPA**		
**Parameter**	**Units**	**Value (RSE%)**	Bootstrap results* median (p2.5^th^—p97.5^th^)		**Value (RSE%)**	Bootstrap results* median (p2.5^th^—p97.5^th^)	
Disposition Parameters							
CL	L/h/70 kg	16.5 (10)	16.0 (13.4–19.6)		11.8 (5)	11.7 (10.5–12.9)	
V_C_	L/70 kg	311 (9)	318.4 (258.8–362.5)		106 (22)	103.7 (48.5–162.5)	
Q	L/h/70 kg	20.5 (12)	21.1 (14.9–26.1)		37.1 (9)	39.1 (18.9–55.3)	
V_P_	L/70kg	56300 (8)	60038.6 (42571.5–70008.1)		800 FIX	-	
Absorption parameters							
K_A_	h^-1^	3.08 (39)	3.42 (2.54–3.62)		1.79 (16)	1.76 (1.25–2.33)	
t_LAG_	h	0.295 (22)	0.308 (0.24–0.35)		0.243 (29)	0.24 (0.18–0.30)	
**Between-subject variability**		**Value (RSE%)**	**SHR**	Bootstrap results* median (p2.5^**th**^**—p97.5**^**th**^**)**	**Value (RSE%)**	**SHR**	Bootstrap results* median (p2.5^**th**^**—p97.5**^**th**^**)**
BSV_CL_	%	57.6 (14)	7%	58.6 (40.4–70.8)	34.9 (11)	8%	35.2 (26.4–41.7)
BSV_Q_	%	68.9 (13)	11%	68.9 (46.3–85.7)	-	-	-
BSV_VC_	%	55.6 (17)	6%	55.5 (32.7–71.5)	133.8 (15)	16%	136.2 (75.5–173.5)
BSV_VP_	%	-	-	-	164.6 (14)	22%	159.8 (88.8–215.0)
Residual error							
Proportional	%	36.6 (12)	5%	37.5 (33.0–42.2)	55.3 (6)	7%	55.5 (50–60)

Abbreviations: CL, whole blood (Tac) or plasma (MPA) clearance; V_C_ and V_P_, central and peripheral volumes of distribution; Q, intercompartmental clearance between compartments; K_A_, absorption rate constant; t_LAG_, lag time; BSV, between-subject variability; RSE, relative standard error; SHR, shrinkage, (-) Not estimated parameter.

* 1000 resamplings were performed.

The observed versus population predicted concentrations and conditional weighted residuals versus time plots displayed in S1 Fig confirmed that the final model adequately described the study population as a whole, without appreciable bias. The low values of ε-shrinkage (5%) allowed for the inspection of the observed versus individual predicted concentrations plots with no evidence of misspecification.

#### Model evaluation

**Fig 1:**
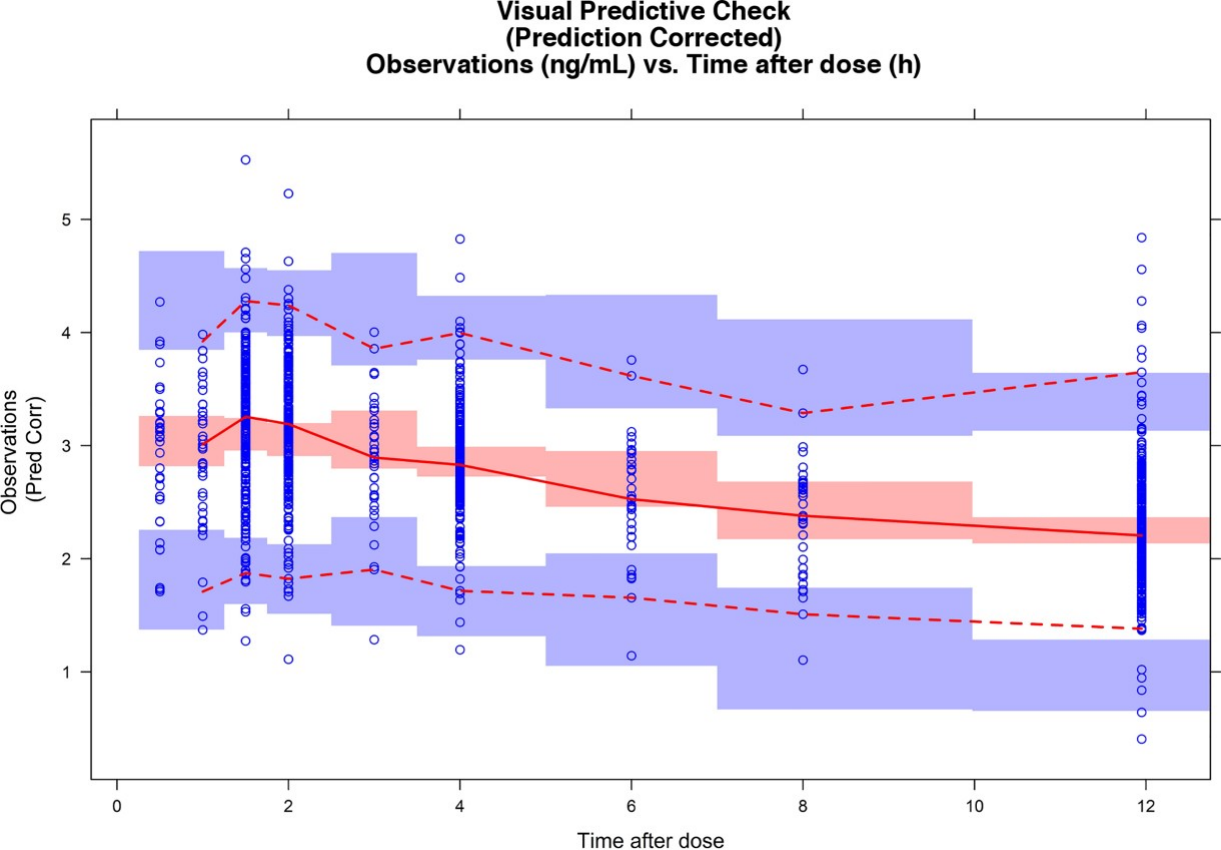
Prediction-corrected visual predictive check for the final tacrolimus population pharmacokinetic model. Blue shaded areas represent the 95% confidence interval of the 97.5th and 2.5th percentiles of the simulated data, and red shaded areas represent the 95% confidence interval of the median of the simulated data. Red dotted lines represent the 97.5th and 2.5th percentiles of the observed data, and the continuous red line is the median of the observed data.

The pcVPC indicated that the model acceptably predicted the median trend of the observed tacrolimus concentrations (Fig 1).The 2.5th, 50th and 97.5th percentiles of the observed data lied within the 95% prediction intervals of the corresponding simulated percentiles, although a slight underprediction was observed around the peak concentration.

The median and 2.5th and 97.5th percentiles obtained by the bootstrap method displayed in Table 4 indicated that all the estimates of population parameters were within the 95% confidence interval (CI), and the maximum relative deviation of the bootstrap median with respect to the population value was 10%.

The distribution of the normalized prediction distribution errors (npde) of the observed data overlapped with the distribution of the errors of the simulated data confirming that the model can predict the median concentration in the overall dataset accurately (S2 Fig).

The stepwise covariate modeling did not identify any covariate as significant; therefore, the results at this stage were considered the final model.

### Mycophenolic acid

A total of 1071 concentration time points from 58 patients were used to develop a two-compartment model with time-lagged first-order absorption and first-order elimination. BSV could be associated only with plasma CL, V_C_ and V_P_. V_P_ was fixed at referenced values taken from the literature (800 L) to adequately estimate the rest of parameters.

Residual error was best described by a proportional error model. The estimated final parameters and their precision, BSV effect on each parameter and η-shrinkage and ε-shrinkage values were acceptable and are listed in Table 4.

Goodness-of-fit plots did not show any remarkable bias as shown in S3 Fig. The observed versus population predicted concentrations plot suggested that the population prediction described adequately the central tendency of the data. No model misspecification was detected related to the structural part or residual error modeling when exploring the remaining plots.

#### Model evaluation

**Fig 2:**
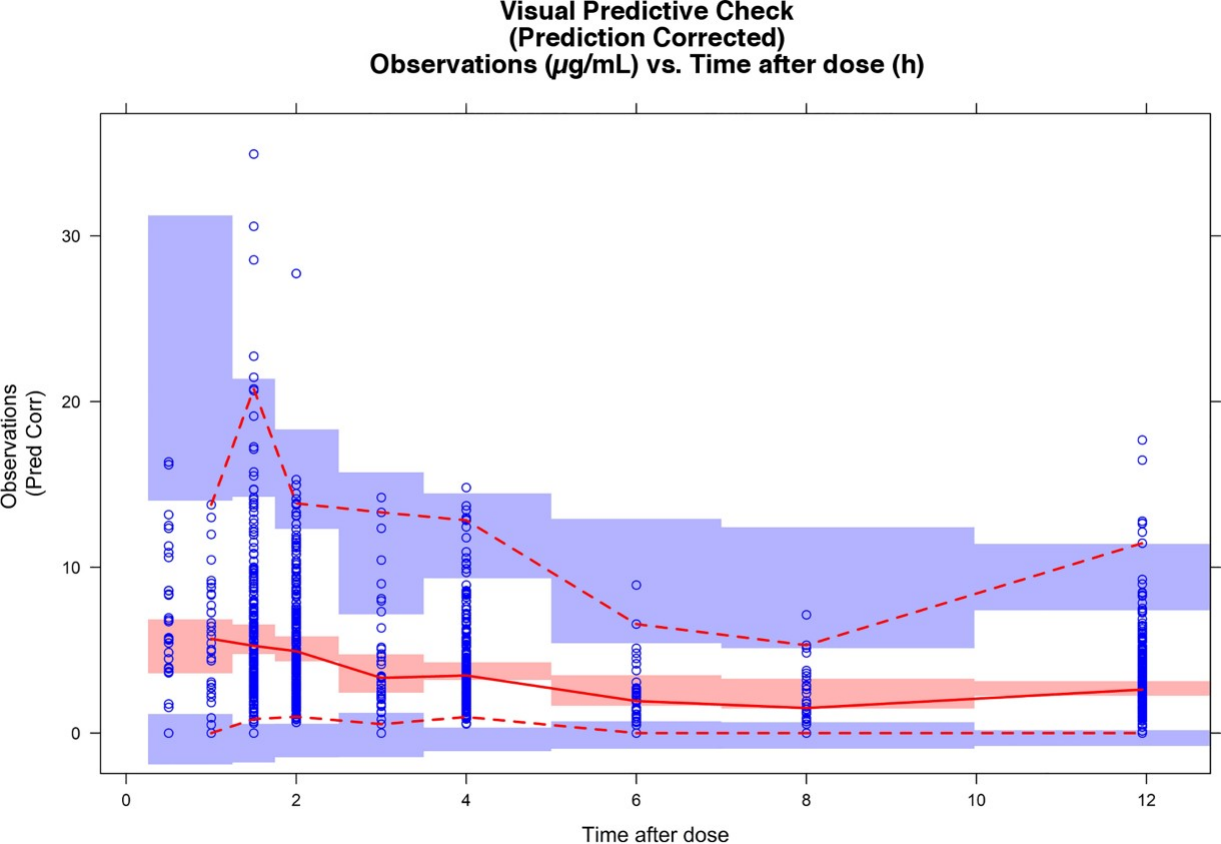
Prediction-corrected visual predictive check for the final mycophenolic acid population pharmacokinetic model. Blue shaded areas represent the 95% confidence interval of the 97.5th and 2.5th percentiles of the simulated data, and red shaded areas represent the 95% confidence interval of the median of the simulated data. Red dotted lines represent the 97.5th and 2.5th percentiles of the observed data, and the continuous red line is the median of the observed data. Dark blue dots are the observation-time points.

The pcVPC indicated that the final model had acceptable predictive value (Fig 2) except around the absorption phase. Overall, the 97.5th, 2.5th and 50th percentiles of the observed data were within the 95% prediction intervals of the corresponding percentiles for the simulated data.

The median and 2.5th and 97.5th percentiles obtained by the bootstrap method are shown in Table 4. All parameters were within the 95% CI, and the relative deviation of the bootstrap median with respect to the population value was lower than 6%.

The distribution of the normalized prediction distribution errors (npde) for the observed data overlapped with the distribution of the errors for the simulated data (S4 Fig).

The base model was considered the final model because none of the tested covariates led to a significant reduction in the MOFV value.

### Logistic regression

The logistic regression model was built with a total of 183 observations/time points from 58 patients. The final model consisted of the linear combination of the baseline effect and the effect of miR155-5p urinary pellet expression. None of the other tested explanatory variables (tacrolimus and MPA estimated mean trough concentrations and cumulative exposures [AUC] and CXCL-10 expression) adequately improved the model so they were not retained. The parameter estimates and RSEs, bootstrap results and final model equations are presented in Table 5.

**Table 5:** Final parameters of the miR155-5p expression -Acute rejection (AR) risk logistic regression model.

Parameter	Values (RSE%)	Bootstrap* mean (P2.5^th^-P97.5^th^)
ß_0_	-5.89 (15)	-5.94 (-9.88 –-4.80)
ß_1_	3.51 (24)	3.45 (2.34–56.72)
	LOGIT(Pi) **=** ß_0_ + ß_1_*miR155-5p	

Abbreviations: ß_0_, Baseline risk of rejection; ß_1_, Slope of explanatory variable effect; RSE%, relative standard error expressed as %.

* 200 resamplings were performed.

#### Logistic regression model evaluation

**Fig 3:**
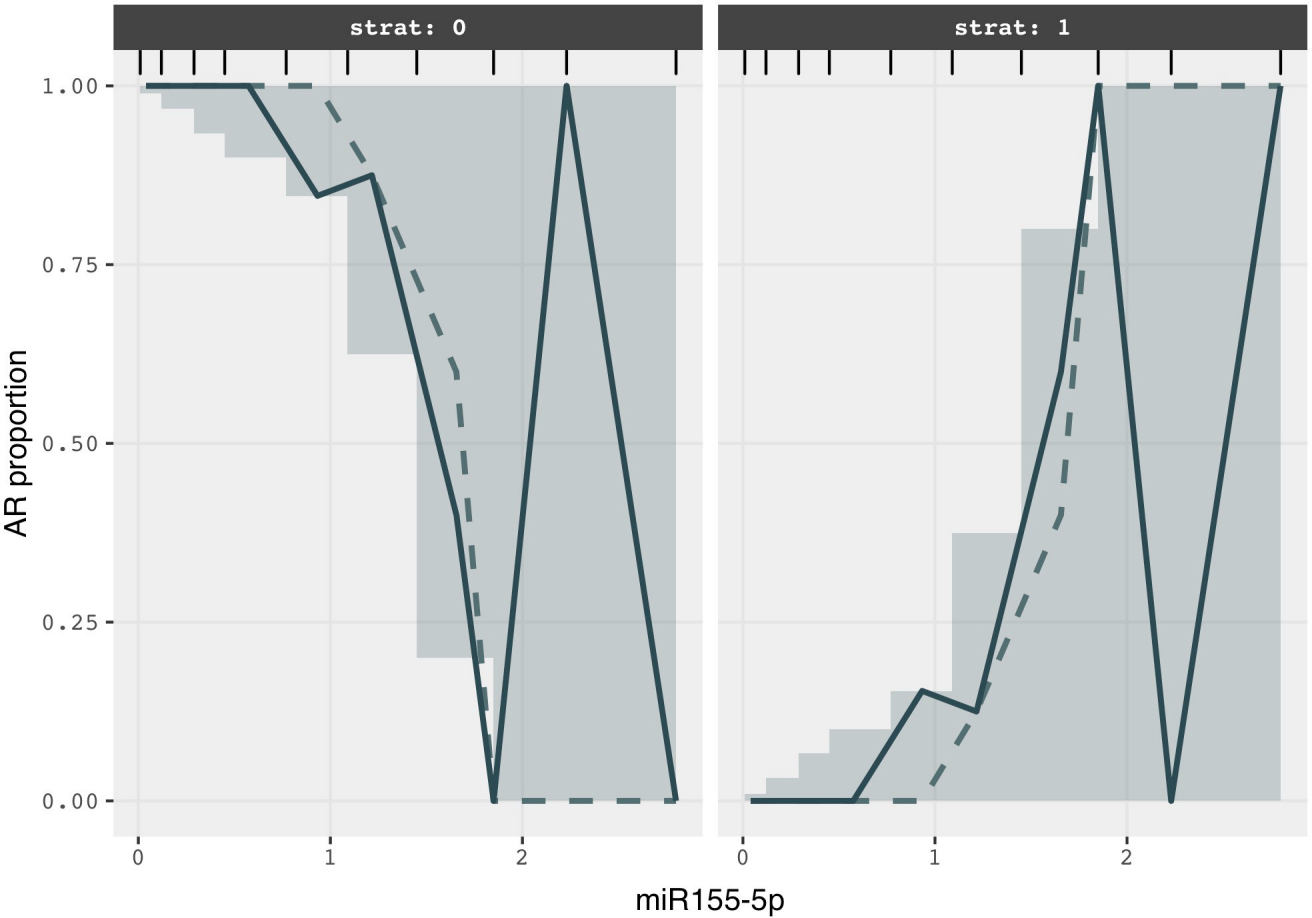
VPC of miR155-5p expression (ΔCt) -AR logistic regression model. Solid line: Median of observed AR proportion versus. miR155-5p expression. Dotted line: Median of predicted AR proportion versus miR155-5p expression; shaded area: 95% prediction interval of AR versus miR155-5p expression.

**Fig 4:**
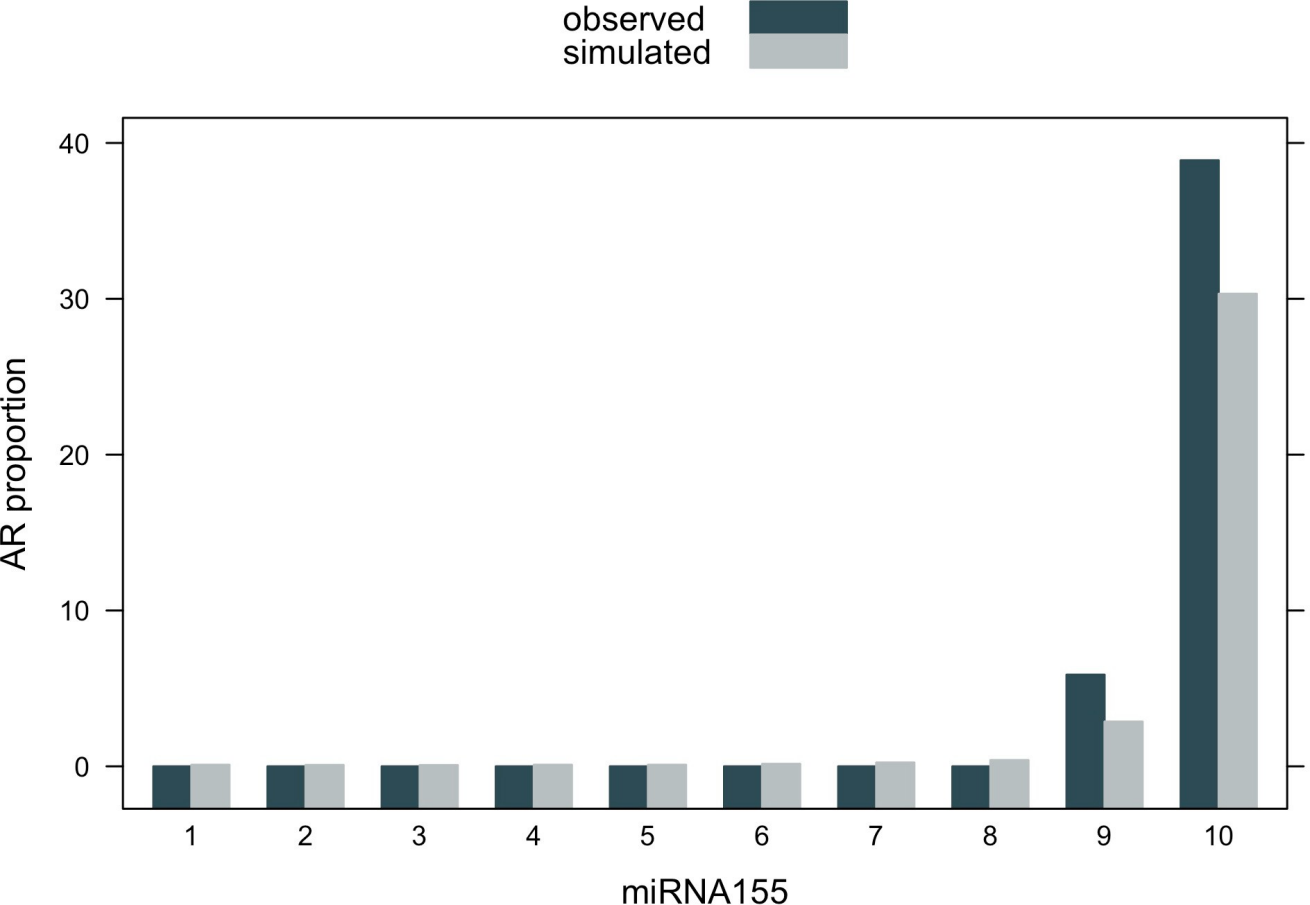
Grouped bar graph of acute rejection proportions versus miR155-5p expression (ΔCt). The green and gray bars are the predicted and observed proportions of rejection occurrence, respectively.

The results of the VPC for the miR155-5p-AR logistic regression model (Fig 3) showed that the simulated and the observed median lines were superimposed except at high biomarker concentrations, likely due to the lack of sufficient data. According to the grouped bar plot in Fig 4, the proportions of rejection occurrence for simulated data at all biomarker concentrations were in agreement with those for observed data.

The results of bootstrap analysis (Table 4) confirmed the accuracy of the final parameter estimates and the stability of the model. The population parameters were all inside the 95% bootstrap CI with a bias lower than 12%.

## Discussion

To our knowledge, this study is the first of adult kidney transplant recipients where a population logistic regression model was developed to identify the potential predictive factors of the risk of AR, using a non-linear mixed effects analysis. Our major aim was to investigate the effect of biomarker expression and ISD exposure on AR. Thus, tacrolimus and MPA population pharmacokinetic models were developed to predict precise exposures. Among the biomarkers evaluated, only urinary miR155-5p expression was predictive of the clinical event. Neither tacrolimus nor MPA individual predicted cumulative exposures (AUC) nor mean observed trough concentrations where identified as predictive factors of AR. This was probably due to the successful implementation of therapeutic drug monitoring overall post-transplant period, even though at the earliest stages (from week 1 to month 1), patients who developed AR showed a tendency toward a lower tacrolimus exposure than those who did not. Similarly occurred with MPA, with comparable exposures between patients experiencing AR and those who did not. These findings were in agreement with those of *Sánchez-Fructuoso et Al*. [36], who found a significant relationship between lower MPA exposures in week 1 and AR occurrence during month 1 post-transplant in kidney recipients through a logistic regression analysis. However, no statistically significant relationship was found in tacrolimus exposure due to similar exposures between patients with AR and patients without. The low number of rejection events could also have contributed to the lack of statistical significance between ISD and AR occurrence of our study.

To our knowledge, this study is the first of adult kidney transplant recipients where a population logistic regression model was developed to identify the potential predictive factors of the risk of AR, using a non-linear mixed effects analysis. Our major aim was to investigate the effect of biomarker expression and ISD exposure on AR. Thus, tacrolimus and MPA population pharmacokinetic models were developed to predict precise exposures. Among the biomarkers evaluated, only urinary miR155-5p expression was predictive of the clinical event. Neither tacrolimus nor MPA individual predicted cumulative exposure (AUC) nor mean observed trough concentrations where identified as predictive factors of AR. The successful implementation of therapeutic drug monitoring overall post-transplant period could explain this lack of predictive ability, even though at the earliest post-transplant stages of the study (from week 1 to month 1), patients who developed AR showed a tendency toward a lower tacrolimus exposure than those who did not. Similarly occurred with MPA, with comparable exposures between patients experiencing AR and those who did not. These findings were in agreement with those of Sánchez-Fructuoso et Al. [36], who found a significant relationship between lower MPA exposures in week 1 and AR occurrence during month 1 post-transplant in kidney recipients, through a logistic regression analysis. However, no significant relationship was found in the case of tacrolimus due to similar exposures between patients with AR and those without. The low number of rejection events may also explain the lack of significance between ISD and AR occurrence of our study.

As aforementioned, miR155-5p was a unique biomarker that predicted the probability of AR in the target population. This finding is in agreement with the results of a previous study by our group [4], where urinary miR155-5p pellet expression significantly increased before an AR episode, demonstrating that miR155-5p expression could be effective as a prognostic biomarker. *Anglicheau* et al. [37] also identified miR155-5p biopsy expression as a diagnostic biomarker for AR in kidney transplant recipients.

The logistic regression model developed in the current study allowed us to confirm these findings and provide a quantitative measure of the effect of changes of miR155-5p on AR risk. Based on this model, an increase of 0.1 units in miR155-5p urinary expression can lead to a 10% increase in AR risk. These results suggest that miR155-5p expression may be considered not only as a diagnostic but also a prognostic biomarker due to the early and progressive increase in its expression before the occurrence of the clinical event.

Unlike miR155-5p expression, the significance between urinary concentration of CXCL-10 and the risk of AR could not be proven. Several published studies have addressed the influence of the urinary concentration of CXCL-10 on the risk of kidney clinical outcomes, such as the risk of tubulitis occurrence in adult transplanted kidney patients [38] or the risk of tubulitis and acute T-cell-mediated rejection (TCMR) in pediatric renal transplantation [39]. In all of these reports, a logistic regression analysis was performed that confirmed the statistical significance of each relationship [39]. These findings suggest that additional studies should be performed with CXCL-10 with a higher number of patients to confirm the previous results.

All of these results support the necessity of monitoring ISDs simultaneously with biomarkers in kidney transplant recipients for a rapid prognostic and diagnostic assessment of rejection, graft outcome and early personalized treatment adjustment, as reported by *Millan* et al. [4].

The implementation of the measurement of urinary miR155-5p expression and CXCL-10 production in routine clinical practice is feasible, considering that the majority of medical centers involved in transplantation use standard methodologies in laboratories. Regarding the frequency of monitoring of these biomarkers after kidney transplantation we and others suggest to monitor them at the 1st week, 2nd week and 1st month post-transplantation (when the incidence of AR is high) and during the process of identifying good candidate patients for ISD treatment minimization (approximately the 3rd month after transplantation). Obviously, the frequency of monitoring of these biomarkers requires further investigation in the context of multicenter randomized clinical trials.

As outlined above, accurate tacrolimus and MPA individual cumulative exposures to be used in the logistic regression model were obtained from the respective population pharmacokinetic models. Although a large number of pharmacokinetic models exist in the literature for tacrolimus and MPA in renal transplant populations, very few of them used SAEM as parameter estimation method. In our case, the efficient handle of full omega blocks of this method provided better estimates of parameter standard errors than first-order conditional estimation methods.

According to most of the previously reported models developed from more than only trough concentrations, the pharmacokinetic profiles of tacrolimus [40,41] and MPA [26,42] were best described by two-compartment models with first-order absorption. Also in agreement with most studies [43], elimination was described by a first-order kinetics. No dose-dependence related with the elimination process or erythrocyte (tacrolimus) or protein binding (MPA) saturation was found in any case.

The estimated CL/F value of tacrolimus was in line with those previously reported (CL/F = 16.5 L/h [70 kg] versus 16.1 L/h [44], 16.5 L/h [45], and 16.3 L/h [40]). Although hematocrit values were not available, the tacrolimus CL/F was close to that expected for a standard hematocrit level of 45% (16.1 L/h), as reported by Storset et al. [41]. Although previous studies have reported the influence of metabolic mass in terms of BSA [46] or free fat mass [44] on tacrolimus clearance, and other authors have estimated empirical allometry exponents for CL/F and VC/F [47], we did not find statistical significance of size covariates on disposition parameters, likely due the scarce number of overweight patients. However, the standard approach used by others [48,49] for scaling body weight by using the fixed exponent values of 0.75 and 1 for flow parameters and distribution volumes, respectively, improved our model predictions.

One of the limitations of our study was the difficulty encountered to properly describe the absorption process in a more physiological way such as the erlang distribution or transit compartment models as others [45]. Unlike other studies [44], the classical lag-time modeling did not allow us to adequately capture peak concentrations, affecting the central compartment distribution volume (Vc) estimated value. The V_C_ of tacrolimus was slightly higher than the values obtained by *Musuamba* et al. (221 L) [50] and *Ogasawara* et al. (214 L) [51], but larger differences were observed compared with studies that described the absorption process by means of transit compartment models [45,52] or those with rich sampling designs during the absorption phase [48].

Ka values (3.08 h-1) also reflected sensibility to the aforementioned points. We found a higher value than those reported by other authors (1.01 h-1 [44], 0.47 h-1 [45], and 0.45 h-1 [40]) but similar to or lower to others that used erlang distribution or transit compartment models [52], regardless of the classical lag-time or erlang distribution models were applied. Basically, differences relied on the success of modeling the absorption phase that in turn depended on the availability of sufficiently informative data.

Higher values of peripheral distribution volume (Vp) were found with respect to those reported in other similar studies. Between-patient variability could not be included in this parameter. Both facts could be attributed to the lack of sufficient data. The Vp prediction is highly dependent on the concentration values associated with the post -distributive phase where the sampling was scarce this probably leading to model overprediction.

The estimated CL/F value for MPA (CL = 11.8 L/h) was lower than that reported previously (28.8 L/h [53]; 25 L/h [40]). Of note, these studies were performed at the earliest phase (9–15 days) of the transplantation period, when the presence of poor renal function could have altered the MPA binding to albumin, resulting in higher clearance values. By contrast, our CL/F value was similar to that calculated from free MPA CLu/F (410 L/h) in the study of *Colom* et al. [54], taking into account the mean MPA unbound fraction found in that study (fu = 0.023) (CL/F = fu·CLu/F = 9.43 L). It is noteworthy that most of the data analyzed in the study of *Colom* et al. were obtained on different occasions during the first year post-transplantation, as was almost the case of the present study (6 months).

Even though the MPA Ka value (Ka = 1.79 h-1) was close to that of previous studies (Ka = 1.79 h-1 versus 1.22 h-1 [26] and 2.3 h-1 [40]), the lack of data hampered a more physiological description of the absorption process, similar to tacrolimus.

Our study presents some other intrinsic limitations. First, the small sample size or low number of patients having AR limited the investigation of tacrolimus exposure and of CXCL-10 as potential predictors of AR. Second, the biomarker analytical determination was conducted in the same laboratory (Pharmacology and Toxicology Laboratory of the Hospital Clinic of Barcelona), and an interlaboratory validation to compare results is desirable. Finally, the study was only performed in Caucasian patients, and additional studies in other races are required.

## Conclusions

A logistic regression model that shows the prognostic capability of miR155-5p for the risk of AR has been developed. This model reinforces the promising results of miR155-5p as a prognostic biomarker for rejection in adult kidney transplants. Indeed, monitoring of miR155-5p could be very useful during routine clinical practice to identify potential patients at high risk of rejection at the early stages of the post-transplant period. This information would allow for the consequent optimization of their treatment. No significant effect of ISD exposures or CXCL-10 on AR was evident, likely due to the low sample size. Therefore, additional multicentric randomized studies are required to confirm the potential of miR155-5p and to further investigate CXCL-10 as prognostic biomarkers of risk of rejection and as useful tools to complement tacrolimus and MPA monitoring.

## Supporting information

S1 Fig Tacrolimus goodness of fit plots. Left upper panel: Observed versus population predicted concentrations (ng/mL). Right upper panel: Observed versus individual predicted concentrations (ng/mL). Left lower panel: Individual weighted residuals versus individual predicted concentrations (ng/mL). Right lower panel: Conditional weighted residuals versus time (h).
(PNG)

S2 Fig Tacrolimus NPDE plots. Left upper panel: Quantile-quantile plot of the npde versus the expected standard normal distribution. Right upper panel: Histogram of the npde with the density of the standard normal distribution overlayed. Left lower panel: Scatterplot of the normalized prediction distribution errors versus time (h). Right lower panel: Scatterplot of the normalized prediction distribution errors versus predicted concentrations (ng/mL).
(PNG)

S3 Fig MPA goodness of fit plots. Left upper panel: Observed versus population predicted MPA concentrations (μg/mL). Right upper panel: Observed versus individual predicted MPA concentrations(μg/mL). Left lower panel: Weighted residuals versus individual predicted MPA concentrations (μg/mL). Right lower panel: Conditional weighted residuals versus time (h).
(PNG)

S4 Fig MPA NPDE plots. Left upper panel: Quantile-quantile plot of the npde versus the expected standard normal distribution. Right upper panel: Histogram of the npde with the density of the standard normal distribution overlayed. Left lower panel: Scatterplot of the normalized prediction distribution errors versus time (h). Right lower panel: Scatterplot of the normalized prediction distribution errors versus predicted concentrations (μg/mL).
(PNG)

S1 Table Tacrolimus PK model dataset. (CSV)

S2 Table MPA PK model dataset. (CSV)

S3 Table Logistic regression model dataset. (CSV)

S1 Appendix Model codes. (DOCX)
