# Supplementary figures and images for: Early prognostic performance of miR155-5p monitoring for the risk of rejection: Logistic regression with a population pharmacokinetic approach in adult kidney transplant patients
Source: PLoS One. 2021 Jan 22;16(1):e0245880. doi: 10.1371/journal.pone.0245880 (PMC7822507; doi:10.1371/journal.pone.0245880)

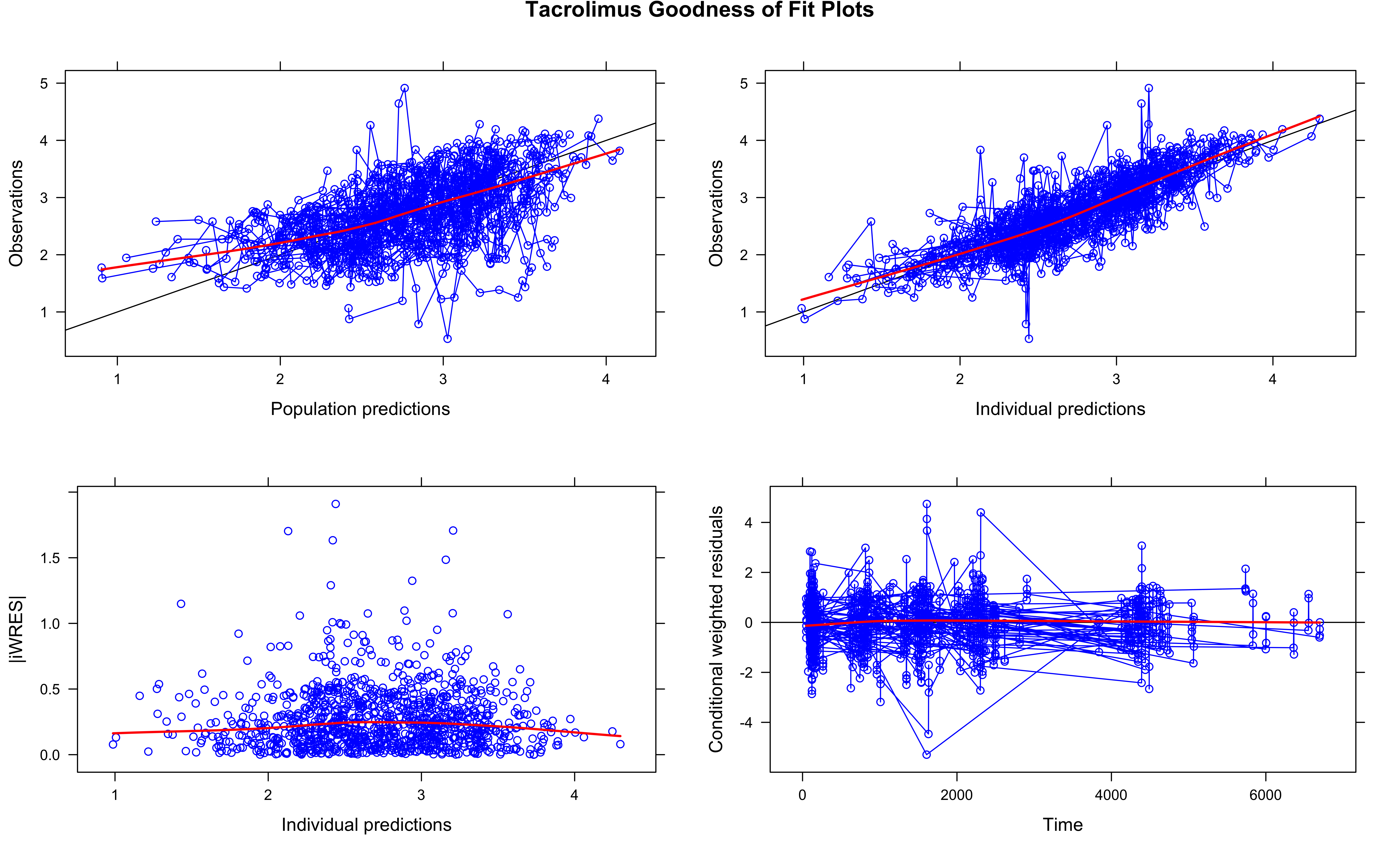

Supplement: S1 Fig — Left upper panel: Observed versus population predicted concentrations (ng/mL). Right upper panel: Observed versus individual predicted concentrations (ng/mL). Left lower panel: Individual weighted residuals versus individual predicted concentrations (ng/mL). Right lower panel: Conditional weighted residuals versus time (h). (PNG) [file pone.0245880.s001.png]

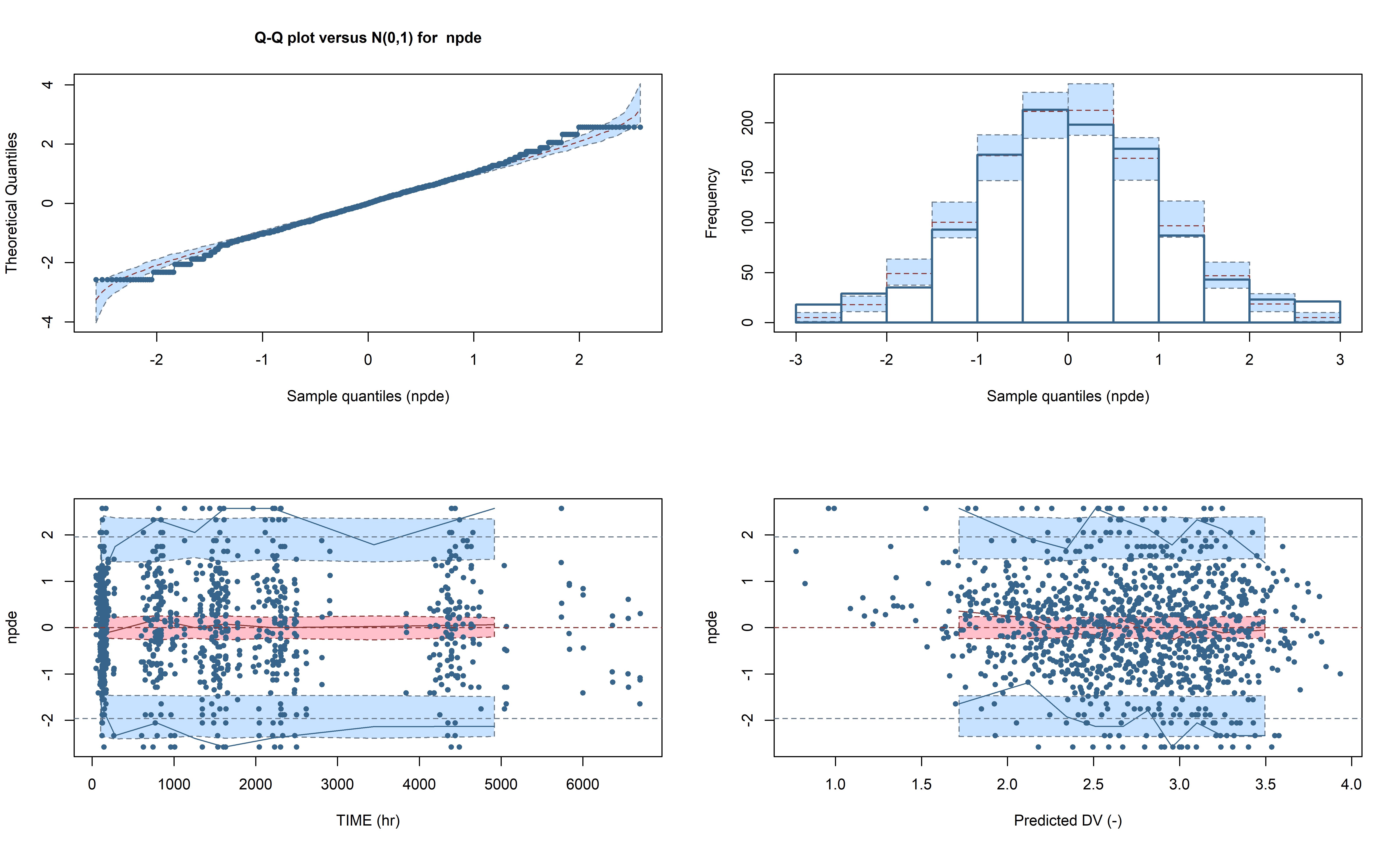

Supplement: S2 Fig — Left upper panel: Quantile-quantile plot of the npde versus the expected standard normal distribution. Right upper panel: Histogram of the npde with the density of the standard normal distribution overlayed. Left lower panel: Scatterplot of the normalized prediction distribution errors versus time (h). Right lower panel: Scatterplot of the normalized prediction distribution errors versus predicted concentrations (ng/mL). (PNG) [file pone.0245880.s002.png]

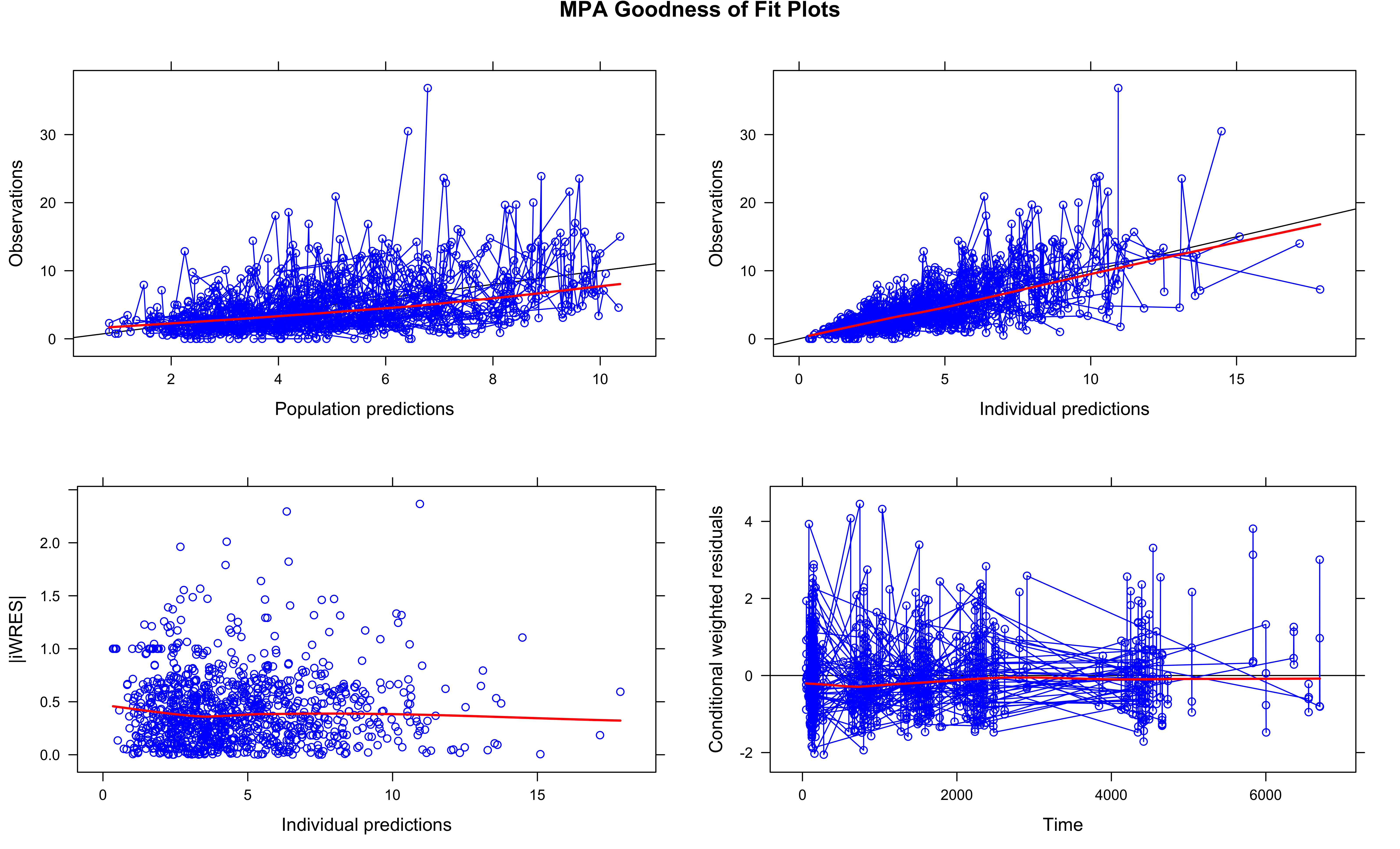

Supplement: S3 Fig — Left upper panel: Observed versus population predicted MPA concentrations (μg/mL). Right upper panel: Observed versus individual predicted MPA concentrations(μg/mL). Left lower panel: Weighted residuals versus individual predicted MPA concentrations (μg/mL). Right lower panel: Conditional weighted residuals versus time (h). (PNG) [file pone.0245880.s003.png]

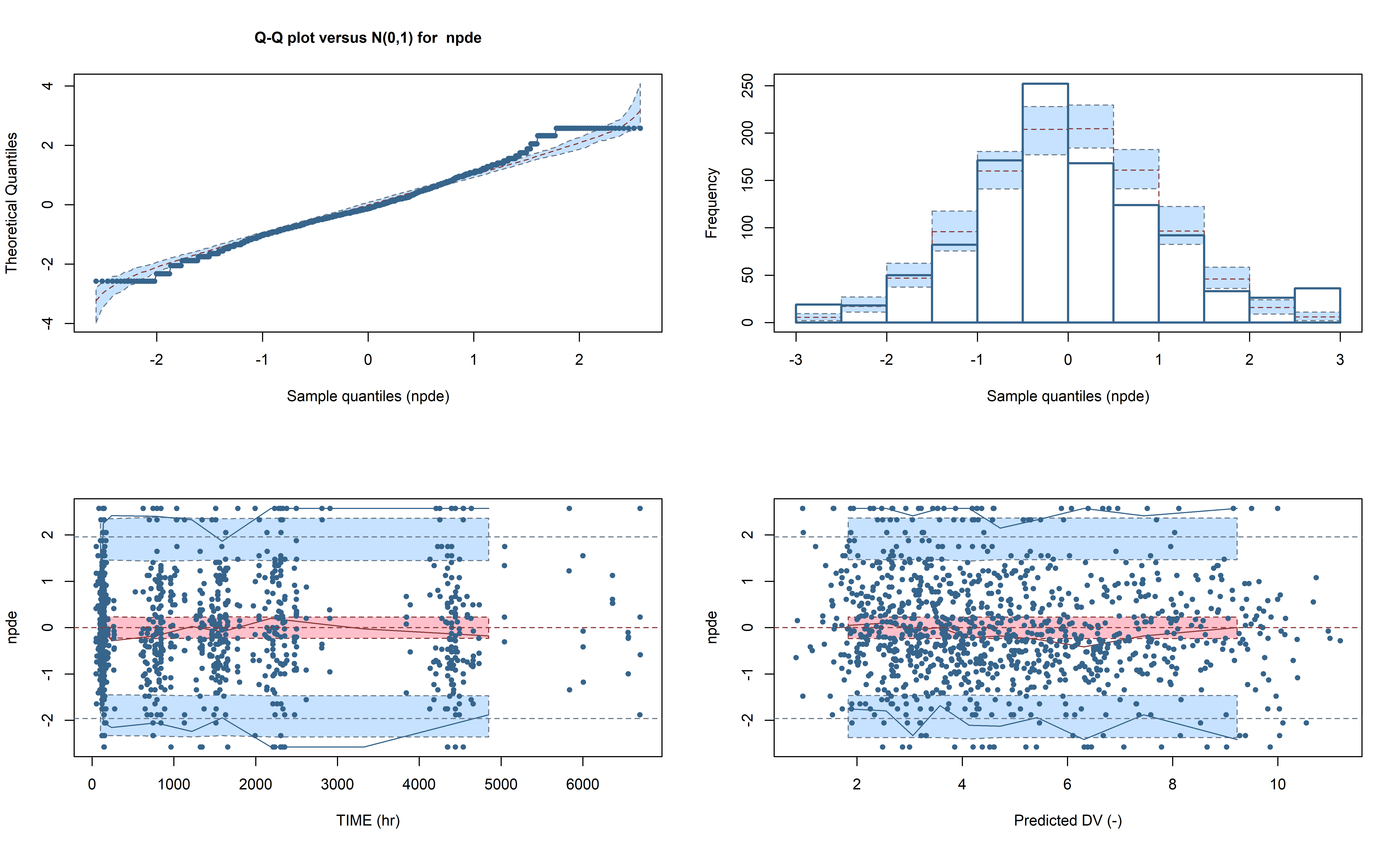

Supplement: S4 Fig — Left upper panel: Quantile-quantile plot of the npde versus the expected standard normal distribution. Right upper panel: Histogram of the npde with the density of the standard normal distribution overlayed. Left lower panel: Scatterplot of the normalized prediction distribution errors versus time (h). Right lower panel: Scatterplot of the normalized prediction distribution errors versus predicted concentrations (μg/mL). (PNG) [file pone.0245880.s004.png]
