# Supplementary material for: Early prognostic performance of miR155-5p monitoring for the risk of rejection: Logistic regression with a population pharmacokinetic approach in adult kidney transplant patients
Source: PLoS One. 2021 Jan 22;16(1):e0245880. doi: 10.1371/journal.pone.0245880 (PMC7822507; doi:10.1371/journal.pone.0245880)
Supplement: S1 Appendix — (DOCX) [file pone.0245880.s008.docx]

# S1 Appendix Models code

## Tacrolimus PK model

$PROBLEM Tacrolimus PK model

;----------------------------------

$INPUT ID TIME TAD AMT DV MDV EVID OCC II ADDL CMT AGE SEX WGT HGH IMC DTY GF IFC AR DAGE CISQ DYL LYM NTA OIFC CMV BKV DM

;----------------------------------

$DATA datalogtacro.csv IGNORE=#;

;

;----------------------------------

$SUBROUTINES ADVAN4 TRANS4

;----------------------------------

$PK

TVCL = THETA(1)

MU_1 = LOG(TVCL)

CL = EXP(MU_1+ETA(1))

TVQ = THETA(2)

MU_2 = LOG(TVQ)

Q = EXP(MU_2+ETA(2))

TVV2 = THETA(3)

MU_3 = LOG(TVV2)

V2 = EXP(MU_3+ETA(3))

TVKA = THETA(4)

MU_4 = LOG(TVKA)

KA = EXP(MU_4+ETA(4))

TVV3 = THETA(5)

MU_5 = LOG(TVV3)

V3 = EXP(MU_5+ETA(5))

TVLT = THETA(6)

MU_6 = LOG(TVLT)

ALAG1= EXP(MU_6+ETA(6))

S2 = V2/1000

TVF1 = 1

F1 = TVF1

YURI = AMT/CL

;----------------------------------

$THETA

(0, 18.5) ;CL

(0, 20.3) ;Q

(0, 334) ;V2

(0, 2.83) ;KA

(0, 48400) ;V3

(0, 0.249) ;tLAG

$OMEGA BLOCK(3)

0.23 ; CL

0.0412 0.586 ; Q

0.197 0.282 0.33 ; V2

$OMEGA 0.01 FIX ; KA

$OMEGA 0.01 FIX ; V3

$OMEGA 0.01 FIX ; tLAG

;----------------------------------

$SIGMA

0.145

;----------------------------------

$ERROR

;--------LOG ERROR--------------

IPRED = -5

IF(F.GT.0) IPRED = LOG(F)

Y = IPRED + EPS(1)

IRES = DV - IPRED

IWRES = IRES

;----------------------------------

$ESTIMATION METHOD=SAEM INTERACTION NBURN=1000 CITER=10, CTYPE=3 CALPHA=0.05 CINTERVAL=10 ISAMPLE=2 NITER=100

PRINT=1 SEED=1234 NOABORT GRD=DDDDD

$ESTIMATION METHOD=IMP INTERACTION EONLY=1 NITER=20 ISAMPLE=300 PRINT=1

NOPRIOR=1 NOABORT

;----------------------------------

$COV PRINT=E UNCONDITIONAL

;----------------------------------

$TABLE ID TIME YURI TAD OCC DV MDV EVID IPRED IWRES CWRES ONEHEADER NOPRINT FILE=sdtab474

$TABLE ID CL V2 Q V3 KA ALAG1 ETA1 ETA2 ETA3 ETA4 ETA5 ONEHEADER NOPRINT FILE=patab474

$TABLE SEX DTY IFC AR OIFC CMV BKV DM ONEHEADER NOPRINT FILE=catab474

$TABLE AGE WGT HGH IMC GF DAGE CISQ DYL LYM ONEHEADER NOPRINT FILE=cotab474

;----------------------------------

## MPA PK model

$PROBLEM MPA PK model

;----------------------------------

$INPUT ID TIME TAD AMT DV MDV EVID OCC II ADDL CMT AGE SEX WGT HGT IMC DTY GF GFC IFC AR DAGE CISQ DYL LYM NTA OIFC CMV BKV DM

$DATA datampa.csv IGNORE=#

;----------------------------------

$SUBROUTINES ADVAN4 TRANS4

;----------------------------------

$PK

;~~~~~~~ COVARIATE MODELING ~~~~~~~

COV = WGT

MCOV = 70

ECOV = (COV/MCOV)**0.75 ; Allometric ALO

DCOV = (COV/MCOV)

;~~~~~~~~~~Mu_modelling~~~~~~~~~~~~

TVCL = THETA(1)

MU_1 = LOG(TVCL)

CL = EXP(MU_1+ETA(1)) * ECOV

TVV2 = THETA(2)

MU_2 = LOG(TVV2)

V2 = EXP(MU_2+ETA(2)) * DCOV

TVV3 = THETA(3)

MU_3 = LOG(TVV3)

V3 = EXP(MU_3+ETA(3)) * DCOV

TVQ = THETA(4)

MU_4 = LOG(TVQ)

Q = EXP(MU_4+ETA(4)) * ECOV

TVKA = THETA(5)

MU_5 = LOG(TVKA)

KA = EXP(MU_5+ETA(5)) * DCOV

TVLT = THETA(6)

MU_6 = LOG(TVLT)

ALAG1= EXP(MU_6+ETA(6))

TVF1=1

F1=TVF1

S2=V2

;----------------------------------

YURI=AMT/CL

$THETA

(0, 12.1) ;CL

(0, 46) ;V2

800 FIX ;V3

(0, 43.1) ;Q

(0, 1.43) ;KA

(0, 0.239) ;tLAG

$OMEGA BLOCK(3)

0.123 ; CL

0.227 1.85 ;V2

0.0677 -0.0932 2.41 ;V3

$OMEGA 0.01 FIX ; Q

$OMEGA 0.01 FIX ; KA

$OMEGA 0.01 FIX ; tLAG

;----------------------------------

$SIGMA

0.31

;----------------------------------

$ERROR

;--------NORMAL ERROR--------------

IPRED = F

IRES = DV-IPRED

W = F

IF(F.EQ.0)W=1

IWRES = IRES/W

Y= IPRED+W*EPS(1)

;-------------SAEM-----------------

$ESTIMATION METHOD=SAEM INTERACTION NBURN=1000 CITER=10, CTYPE=3 CALPHA=0.05 CINTERVAL=10 ISAMPLE=2 NITER=100

PRINT=1 SEED=1234 NOABORT GRD=DDDDD

$ESTIMATION METHOD=IMP INTERACTION EONLY=1 NITER=20 ISAMPLE=300 PRINT=1

NOPRIOR=1 NOABORT

;----------------------------------

$COV MATRIX=R PRINT=E UNCONDITIONAL

;----------------------------------

$TABLE ID TIME YURI TAD OCC DV MDV EVID IPRED IWRES CWRES ONEHEADER NOPRINT FILE=sdtab466

$TABLE ID CL V2 Q V3 KA ETA1 ONEHEADER NOPRINT FILE=patab466

$TABLE SEX DTY IFC AR OIFC CMV BKV DM ONEHEADER NOPRINT FILE=catab466

$TABLE AGE WGT HGT IMC GF DAGE CISQ DYL LYM ONEHEADER NOPRINT FILE=cotab466

;----------------------------------

## miR155-5p Logistic regression

$PROB miR155-5p Logistic regression

$INPUT ID TIME TAD OCC AR=DV M155 AGE SEX WGT HGT IMC DTY GF GFC IFC DAGE CISQ DYL DYLC LYM NTA OIFC CMV BKV DM

$DATA datarlg4.csv IGNORE=#

IGNORE = (M155.EQ.9999)

IGNORE = (M155.EQ.0)

$PRED

EV = M155

B0 = THETA(1)

B1 = THETA(2)*EV

A1 = B0 + B1 + ETA(1)

;------LOGIT OPERATIONS------

C1=EXP(A1)

P1=C1/(1+C1)

PA=1-P1

IF(DV.EQ.1) THEN

Y=P1

ELSE

Y=PA

ENDIF

;-------------------------

$THETA

(-7) ;B0

(4) ;B1

$OMEGA

0 FIX ; B0

;-- SIM ------------------------------------------------------------------------------------------------

;$SIM (12345) (54321 UNI) ONLYSIM NOP NSUB=1000

;-- EST ------------------------------------------------------------------------------------------------

$ESTIMATION METHOD=1 LIKELIHOOD LAPLACE MAX=3000 PRINT=1 NOABORT

;-- TABLES ------------------------------------------------------------------------------------------

$TABLE ID TIME OCC DV P1 Y M155 I10 NOPRINT FILE=sdtab97

$TABLE ID TIME AUCT CT FILE=cotab97

$TABLE ID TIME IFC CMV BKV FILE=catab97

$TABLE ID TIME B0 B1 ETA(1) FILE=patab97
